# Decreased Ebola Transmission after Rapid Response to Outbreaks in Remote Areas, Liberia, 2014

**DOI:** 10.3201/eid2110.150912

**Published:** 2015-10

**Authors:** Kim A. Lindblade, Francis Kateh, Thomas K. Nagbe, John C. Neatherlin, Satish K. Pillai, Kathleen R. Attfield, Emmanuel Dweh, Danielle T. Barradas, Seymour G. Williams, David J. Blackley, Hannah L. Kirking, Monita R. Patel, Monica Dea, Mehran S. Massoudi, Kathleen Wannemuehler, Albert E. Barskey, Shauna L. Mettee Zarecki, Moses Fomba, Steven Grube, Lisa Belcher, Laura N. Broyles, T. Nikki Maxwell, Jose E. Hagan, Kristin Yeoman, Matthew Westercamp, Joseph Forrester, Joshua Mott, Frank Mahoney, Laurence Slutsker, Kevin M. DeCock, Tolbert Nyenswah

**Affiliations:** Centers for Disease Control and Prevention, Atlanta, Georgia, USA (K.A. Lindblade, J.C. Neatherlin, S.K. Pillai, K.R. Attfield, D.T. Barradas, S.G. Williams, D.J. Blackley, H.L. Kirking, M.R. Patel, M. Dea, M.S. Massoudi, K. Wannemuehler, A.E. Barskey, S.L. Mettee Zarecki, S. Grube, L. Belcher, L.N. Broyles, T.N. Maxwell, J.E. Hagan, K. Yeoman, M. Westercamp, J. Forrester, J. Mott, F. Mahoney, L. Slutsker, K.M. DeCock);; Ministry of Health and Social Welfare, Monrovia, Liberia (F. Kateh, T.K. Nagbe, E. Dweh, M. Fomba, T. Nyenswah)

**Keywords:** Ebolavirus Zaire, disease outbreaks, epidemics, epidemiology, Liberia, patient isolation, basic reproduction number, survival, case-fatality rate, viruses, Ebola

## Abstract

We measured the reproduction number before and after interventions were implemented to reduce Ebola transmission in 9 outbreaks in Liberia during 2014. We evaluated risk factors for secondary cases and the association between patient admission to an Ebola treatment unit (ETU) and survival. The reproduction number declined 94% from 1.7 (95% CI 1.1–2.6) to 0.1 (95% CI 0.02–0.6) after interventions began. The risk for secondary infections was 90% lower for patients admitted to an ETU (risk ratio 0.1, 95% CI 0.04–0.3) than for those who died in the community. The case-fatality rate was 68% (95% CI 60–74), and ETU admission was associated with a 50% reduction in death (hazard ratio 0.5, 95% CI 0.4–0.8). Isolation and treatment of Ebola patients had the dual benefit of interrupting community transmission and improving survival.

## Abstract 2

Basic interventions and community acceptance can result in rapid control of outbreaks.

The current Ebola virus disease (Ebola) epidemic in West Africa, caused by the Zaire strain, is the largest in history; >27,000 cases have been reported since Ebola was detected in Guinea in March 2014. Ebola in humans often begins with a nonspecific febrile illness and can progress to gastrointestinal symptoms, hemorrhage, sepsis, multiorgan failure, and death. Person-to-person transmission typically occurs through close contact with the blood or body fluids of a symptomatic infected person during care at home or in health care facilities or during traditional funeral rites (*1*). Case-fatality rates (CFRs) typically are high (68%–90%) for the Zaire strain (*2*,*3*). Although no cure exists for Ebola, supportive therapy, including intravenous fluids and electrolyte replacement, has been found to increase survival (*4*). Transmission of the virus can be reduced by isolation of patients, implementing infection control procedures while providing patient care, and avoiding direct contact with recently deceased persons (*1*).

The first case of Ebola in Liberia occurred in Lofa County in March 2014. The virus spread to the capital, Monrovia, by the end of May and to 10 of 15 counties by August 2014 (*5*). During July–December 2014, several Ebola outbreaks were detected in remote rural areas of Liberia, largely initiated by patients traveling from Monrovia (*6*). Because of difficult access, suboptimal medical care, limited telecommunications coverage, and low levels of health education in these areas, introduction of the Ebola virus led to several complex outbreaks requiring a rapid and coordinated public health response to halt transmission. Systematic prospective investigations of 9 of these outbreaks, all outside of Montserrado County, by the Ministry of Health and Social Welfare (MOHSW), the US Centers for Disease Control and Prevention, the World Health Organization (WHO), and other partners provided an opportunity to characterize Ebola transmission and measure the association among implementation of interventions, transmission, and survival.

## Methods

### Outbreak Investigations and Response

An Ebola outbreak was defined as >2 cases in a community within a 21-day period. Each remote community with an outbreak presented different challenges to an effective public health response, but as part of the interventions implemented in each outbreak, symptomatic persons were immediately isolated (through self-isolation in the home or transfer to an Ebola treatment unit [ETU]), and their contacts were identified and monitored. In 2 communities, challenges in accessibility combined with the presence of severely ill community members required the rapid establishment of temporary isolation and treatment facilities in the community. In the other communities, symptomatic residents were provided transport to an ETU after they traveled by foot to the nearest point accessible by an ambulance. Other interventions provided to the affected communities included promotion of Ebola prevention messages and training in safe and hygienic burials.

Standard MOHSW case investigation forms were completed for all case-patients through interviews with the case-patients or proxies. Case-patient status at the time of report was classified as alive or deceased. During the outbreak investigations, epidemiologists developed transmission chains retrospectively by identifying the source-patients for known cases and linking them through chains of infection to the index case. Prospectively, additional cases were identified through monitoring of contacts and active case finding (*7*). Cases were classified as suspected or probable on the basis of MOHSW guidelines, adapted from WHO-recommended case definitions (*7*). Confirmed cases were identified by laboratory diagnosis of Ebola using real-time reverse transcription PCR of a venous blood sample or a postmortem buccal swab.

During field investigations, the likely source-patient was identified for each case-patient through interviews with the case-patient or proxies; when multiple source-patients were possible (e.g., during intrahousehold transmission), the source-patient was considered missing for analysis purposes. The number of secondary cases generated by each case was determined from the transmission chain dendrograms when a clear epidemiologic link existed between a source-patient and >1 successive cases.

Missing information from case investigations was supplemented by manual searches of ETU and laboratory databases. Date of patient recovery was recorded from ETU databases and defined as the date of discharge; ETUs routinely discharged patients after symptoms had resolved and 1–3 blood samples tested negative for Ebola virus. For case-patients who survived their illness in the community without admission to an ETU, the date of recovery was the first date on which investigators could verify that the person was no longer symptomatic.

### Statistical Analysis

The minimum incubation period was calculated as the number of days between last exposure to the source-patient and symptom onset of the case-patient. The clinical serial interval was the number of days between dates of symptom onset of successive cases linked in a transmission chain.

We compared categorical variables using a χ^2^ test and changes in categorical variables over time using a Cochran-Armitage test for trend. Cases in each outbreak were classified as occurring before or after public health interventions began in the community based on date of symptom onset. The reproduction number *R* (i.e., number of cases in an uninfected population that 1 case generates during its infectious period) was calculated as the mean number of secondary infections from cases that occurred before (*R*_0_) and after (*R*_t_) interventions began in the community. We computed 95% CIs for reproduction numbers by using a negative binomial model accounting for the extra correlation from data clustered by community. Percentage reduction in reproduction number was calculated as (*R*_0_ − *R*_t_)/*R*_0_ × 100%.

We calculated risk ratios (RRs) for infection of >1 secondary cases using generalized estimating equations with a log-binomial distribution (*8*). The extra correlation from clustering by community was accounted for by using an exchangeable correlation structure. RRs and 95% CIs were calculated and a Score χ^2^ test, adjusted for small sample sizes (*9*), of p<0.05 determined the statistical significance of variables (*10*). Percentage reduction in transmission was calculated as (1 − RR) × 100%. We estimated survival distributions by case-patient admission to an ETU using a Kaplan-Meier curve accounting for the number of days between symptom onset and ETU admission and clustering by community. The association between admission to an ETU and survival was evaluated with a Cox proportional-hazards model by using the survival package in R v.3.1.1 (*11*). Admission to an ETU was a binary time-dependent variable entered into the model by using the counting process method. Survival was defined as the number of days from the date of symptom onset until death and was censored at the time of discharge from an ETU or recovery in the community. Robust SEs were used to calculate the 95% CI of the hazard ratio (HR) to account for correlated observations within communities. The assumption of proportional hazards was assessed with the use of Schoenfeld residuals. Percentage reduction in survival was calculated as (1 − HR) × 100%.

### Ethical Considerations

This investigation was conducted as part of the Ebola public health response in Liberia. It was not considered to be human subjects research, in accordance with the US federal human subjects protection regulations and the US Centers for Disease Control and Prevention’s Guidelines for Defining Public Health Research and Public Health Non-Research.

## Results

**Figure 1:**
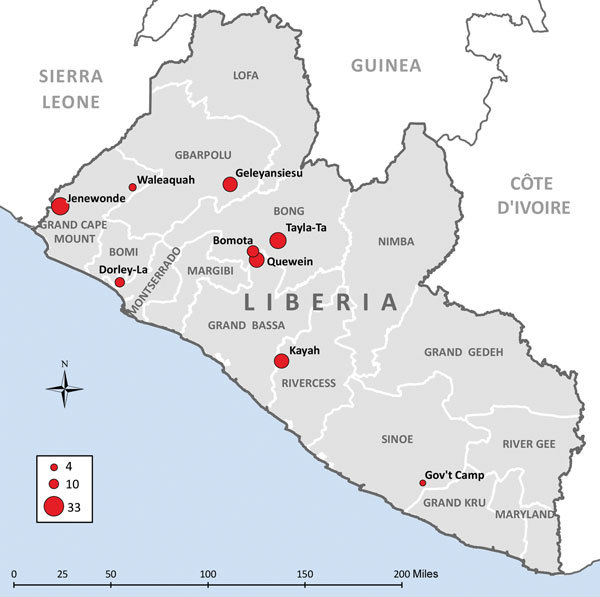
Communities in remote rural areas where Ebola virus disease outbreaks occurred, Liberia, August–December 2014. Size of red dot indicates number of cases.

Fifteen outbreaks of Ebola occurred in remote areas of Liberia during July–December 2014 (*6*); 9 had transmission chains linking most cases to a source-patient and are included in this report (Figure 1; Table 1). The time when MOHSW was notified of an outbreak to the first day of public health intervention in the community was a median of 32 days (range 9–58 days) (Technical Appendix Figures 1, 2).

**Table 1:** Characteristics of Ebola virus disease outbreaks, Liberia, August–December 2014

Community, county	Estimated population	Index case-patient		No. cases	Days to intervention	No. transmission generations	Outbreak duration, d	% Confirmed	% Isolated and treated	CFR, %
		Date of symptom onset	Origin							
Jenewonde, Grand Cape Mount	800	Aug 28	Monrovia	35	58	7	97	40	31	91
Dorley-La, Bomi	301	Sep 16	Monrovia	10	44	3	52	40	40	100
Geleyansiesu, Gbarpolu	800	Sep 18	Margibi and unknown	22	33	4	41	82	68	73
Bomota, Bong	397	Oct 12	Unknown	14	32	3	39	86	86	50
Government Camp, Sinoe	6,200	Oct 13	Monrovia	4	24	1	7	75	75	50
Quewein, Grand Bassa	371	Oct 14	Monrovia	24	43	4	58	75	54	61*
Kayah, Rivercess	5,000	Oct 16	Monrovia	22	26	2	27	59	41	59
Tayla-ta, Bong	500	Oct 24	Monrovia	28	14	1	22	96	96	50
Waleaquah, Grand Cape Mount	700	Nov 20	Monrovia (both index case-patients)	6	9	1	12	100	100	50
*One case-patient died from an accident and is not included in the calculation of CFR. CFR, case-fatality rate.										

### Description of Case-Patients

A total of 165 persons had an illness meeting the case definition for Ebola. Ninety-one (55%) patients were female, and median age was 33 years (range 15 days–84 years) for the 161 patients for whom age was known (Table 2). The most common symptoms reported during the case investigations were fever (92%), intense fatigue (86%), weight loss (63%), and muscle pain (58%) (Table 2).

**Table 2:** Characteristics of 165 case-patients with Ebola virus disease in 9 outbreaks in remote rural areas, Liberia, August–December 2014

**Characteristic**	No. (%) patients*
**Female sex**	91 (55)
**Age, y†**	
** 0–14**	33 (21)
** 15–19**	12 (7)
** 20–29**	25 (16)
** 30–39**	30 (19)
** 40–49**	26 (16)
** 50–59**	18 (11)
**>60**	17 (11)
**Type of case**	
** Confirmed**	115 (70)
** Probable**	38 (23)
** Suspected**	12 (7)
**Outcomes**	
** Admitted to Ebola treatment unit**	100 (61)
** Died**	52 (51)
** Recovered**	49 (49)
** Not admitted to Ebola treatment unit**	64 (39)
** Died**	59 (92)
** Recovered**	4 (6)
Died accidentally	1 (2)
**Symptom**	
** Fever**	70 (92)
** Intense fatigue**	64 (86)
** Weight loss**	24 (63)
** Muscle pain**	40 (58)
** Headache**	31 (41)
** Vomiting/nausea**	26 (37)
** Difficulty breathing**	29 (39)
** Abdominal pain**	27 (36)
** Diarrhea**	26 (35)
** Hiccups**	25 (34)
** Difficulty swallowing**	7 (15)
** Unexplained bleeding**	2 (3)

*Percentages are proportions of non-missing data. **†Case-patients were a median of 33 years of age (range 15 days–84 years).**

**Figure 2:**
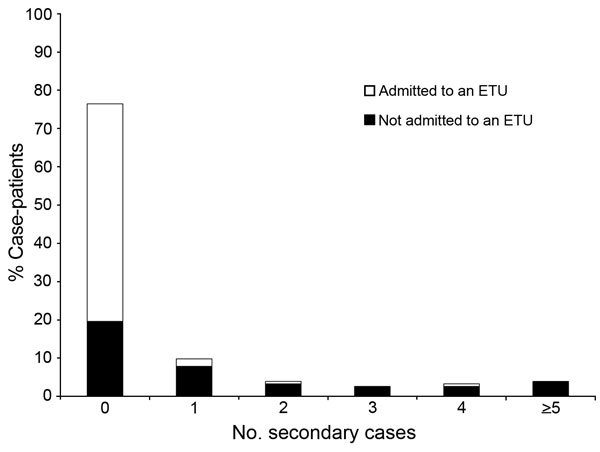
Distribution of Ebola virus disease case-patients by number of secondary cases generated and admission to an Ebola treatment unit (ETU) in remote rural areas of Liberia, August–December 2014.

Ebola was laboratory-confirmed in 115 (70%) case-patients, and 112 (68%) died; however, because 1 death resulted from drowning, the CFR was 68% (95% CI 60%–74%). One hundred (61%) case-patients were isolated and treated in an ETU. Of those admitted to an ETU, 51% (95% CI 41%–61%) died, compared with 94% (95% CI 85%–98%) of the 63 (38%) case-patients not admitted to an ETU (p<0.0001) (Figure 2). Four (6%) case-patients (3 with laboratory-confirmed Ebola) were known to have survived their illness in the community without medical attention.

### Time Intervals

The median minimum incubation period was 8 days (mean 8.4 ± SD 3.7 days) (Technical Appendix Figure 2, panel A), and the median clinical serial interval was 15 days (mean 15.1 ± SD 4.5 days) (Technical Appendix Figure 2, panel B). Time intervals for patient outcomes and length of ETU stay can be found in Technical Appendix Figures 3 and 4. The intervals between symptom onset of individual case-patients and the start of interventions in each community are presented in Technical Appendix Figure 5.

### Secondary Cases

We identified the source-patient of 138 (90%) of the 155 nonindex cases. The number of secondary cases was determined for 157 (95%) case-patients. The proportion of cases for which the source-patient was identified did not differ before and after investigation (90% and 89%, respectively, p = 0.89). Most (76%) case-patients generated no secondary cases (median 0, mean 0.9, range 0–27) (Figure 2). Case-patients who died in the community generated 93% of the secondary cases, whereas case-patients admitted to an ETU generated 7%, and case-patients who survived their illness in the community generated <1%. Six case-patients, all of whom died in the community, infected 55% of the secondary case-patients identified.

The risk for secondary infections was lower for children <15 years of age (RR 0.2, 95% CI 0.1–0.5) and adults 40–49 (RR 0.2, 95% CI 0.1–0.5) than for adults >60 years of age, but age overall was not statistically associated with infection of secondary cases (p = 0.12) (Table 3). Compared with case-patients who died in the community, case-patients admitted to an ETU were associated with a 90% lower risk for infection of secondary cases (RR 0.1, 95% CI 0.04–0.3) (Table 3). Case-patients with symptom onset after interventions began in their community were significantly less likely to generate a secondary case than were case-patients who became ill before interventions started (RR 0.1, 95% CI 0.02–0.8).

**Table 3:** Risk factors for >1 secondary Ebola virus disease cases in outbreaks in remote rural areas, Liberia, August–December 2014

Characteristic	>1 Secondary cases, no./total (%), n = 156	Risk ratio (95% CI)	p value
Sex			0.45
F	21/85 (25)	1.2 (0.8–1.7)	
M	16/71 (23)	Referent	
Age, y			0.12
0–14	3/32 (9)	0.2 (0.1–0.5)	
15–19	3/8 (38)	1.0 (0.4–2.5)	
20–29	5/25 (20)	0.6 (0.3–1.2)	
30–39	8/30 (27)	0.6 (0.3–1.0)	
40–49	5/24 (21)	0.2 (0.1–0.5)	
50–59	5/17 (29)	0.8 (0.4–1.5)	
>60	7/17 (41)	Referent	
Outcome			0.02
Admitted to Ebola treatment unit	5/95 (5)	0.1 (0.04–0.3)	
Recovered in the community	1/4 (25)	0.5 (0.1–3.3)	
Died in the community	31/57 (54)	Referent	
Timing of case			0.02
Before intervention	30/77 (39)	Referent	
After intervention	5/75 (7)	0.1 (0.02–0.8)	

**Figure 3:**
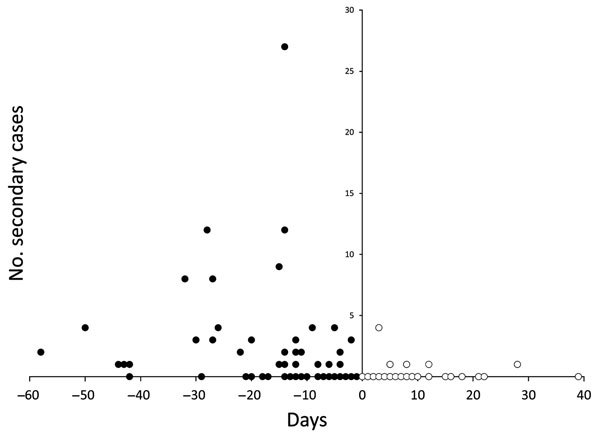
Number of Ebola virus disease secondary cases generated by case-patients, by time from symptom onset to start of interventions, in remote rural areas of Liberia, August–December 2014. Black circles indicate cases that occurred before the start of interventions (day 0); white circles indicate cases that occurred after interventions started.

During the preintervention period, the number of secondary cases ranged from 0 to 27, and the reproduction number was 1.7 (95% CI 1.2–2.6). After interventions began, the number of secondary cases was 0–4, and the reproduction number declined to 0.1 (95% CI 0.02–0.6) (Figure 3), a 94% decrease in transmission.

### CFRs Over Time and By Age

**Figure 4:**
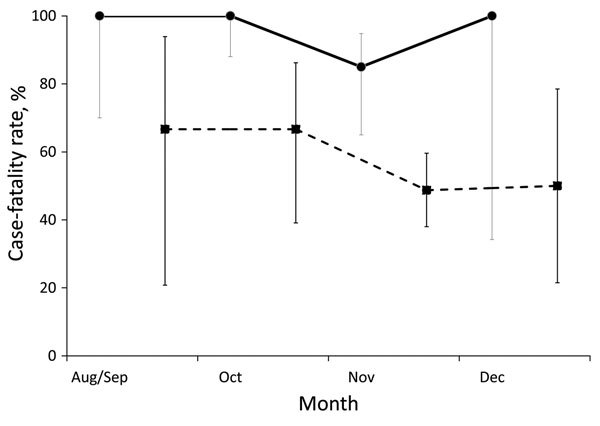
Case-fatality rates for Ebola virus disease, by case-patient admission to an Ebola treatment unit (ETU) and month of symptom onset, in remote rural areas of Liberia, August–December 2014. Dashed lines indicate case-patients admitted to ETU; solid lines indicate patients not admitted to ETU. Error bars indicate 95% CIs.

Overall CFR declined significantly over time (p = 0.002), from 92% in August and September to 60% in December (Figure 4). The CFR for case-patients admitted to an ETU was 67% in August and September and 50% in December (95% CI 22%–78%), but there was no significant trend in CFR for case-patients admitted to an ETU (p = 0.38).

**Figure 5:**
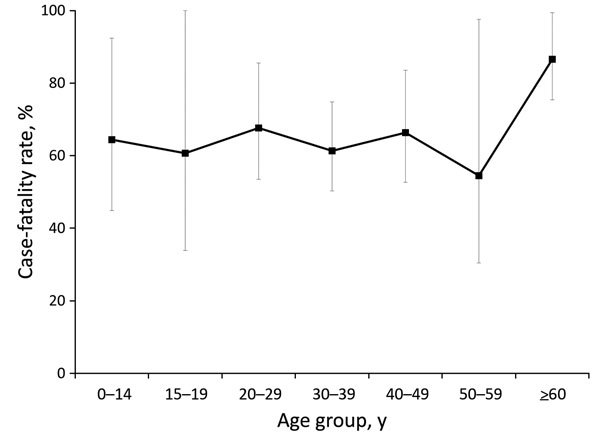
Case-fatality rates for Ebola virus disease, by case-patient age group, in remote rural areas of Liberia, August–December 2014. Error bars indicate 95% CIs.

CFR by age group ranged from 88% for case-patients >60 years of age to 56% for those 50–59 years (Figure 5). Overall, however, CFR by age did not differ significantly by age group (p = 0.67).

### Survival Analysis

**Figure 6:**
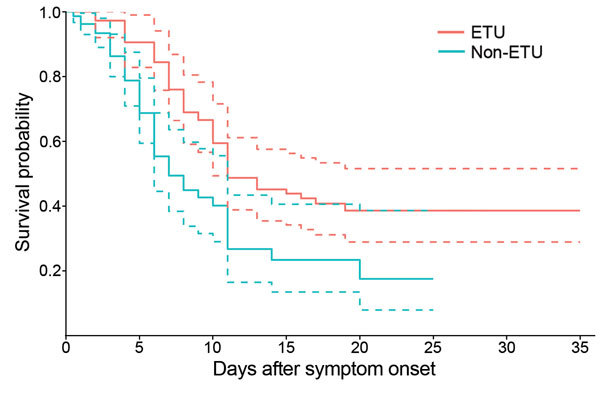
Kaplan-Meier survival curves comparing survival by admission to an Ebola treatment unit (ETU) in remote rural areas of Liberia, August–December 2014. Dashed lines indicate 95% CIs.

The Kaplan-Meier estimate of the median time to death from symptom onset for case-patients who reached an ETU was 11 days (95% CI 10–∞ days), compared with 8 days (95% CI 6–11 days) for case-patients not admitted to an ETU (Figure 6; Schoenfeld residuals in Technical Appendix Figure 6). The Cox proportional-hazards model did not find the HR to vary by sex (p = 0.37) or age (p = 0.27), but admission to an ETU was associated with a 50% reduction in risk for death (HR 0.5, 95% CI 0.3–0.8, p = 0.04) (Table 4); this model was unadjusted because no other variables were found to be associated with survival in univariate modeling.

**Table 4:** Hazard ratios for survival during outbreaks of Ebola virus disease in remote rural areas, Liberia, August**–**December 2014

Characteristic	**Hazard ratio (95% CI)**	**p value**
Sex		0.37
F	0.8 (0.5–1.3)	
M	Referent	
Age, y		0.27
0–14	Referent	
15–19	0.9 (0.2–4.2)	
20–29	0.8 (0.4–1.8)	
30–39	0.8 (0.4–1.7)	
40–49	0.8 (0.3–1.8)	
50–59	0.5 (0.2–1.9)	
>60	1.5 (0.7–3.4)	
Ebola treatment unit		0.04
No	Referent	
Yes	0.5 (0.3–0.8)	

## Discussion

We found a 94% decrease in Ebola transmission after initiation of community interventions in 9 outbreaks in remote rural areas of Liberia during August–December 2014. Isolation and treatment of case-patients in an ETU was associated with a 90% lower risk for secondary cases than those who died in the community and with a 50% lower risk for death than those not admitted to an ETU. Liberia was declared free of Ebola on May 9, 2015 (*12*); however, 3 new cases were identified in July 2015 (*13*).

Although ETUs are a critical intervention to reduce Ebola transmission in the community (*14*), treatment of Ebola case-patients is limited to supportive care, for which the efficacy and effectiveness remain unknown. The WHO Response Team found that the CFR was lower for hospitalized Ebola patients in West Africa (64%) than for all patients (71%) but recognized that this finding could result from multiple possible case ascertainment biases rather than a direct effect of ETU admission (*15*). The potential for bias also cannot be discounted for this study. If mild illness was less likely to be identified during community investigations or if severely ill patients were less likely to reach an ETU, our estimate of the impact of ETU admission on survival might be an overestimate. The data used in this analysis were collected prospectively by teams of experienced epidemiologists and local public health authorities. Complete transmission chains were developed, reducing the chances that cases were missed. In addition, robust statistical analyses adjusting for the number of days from symptom onset to ETU admission were used to account for the possibility that early treatment could increase the chances of survival or that a longer period before admission could introduce a survivor bias. With all the caveats inherent to an observational study, we believe that these data provide evidence that ETU admission improved the chances for patient survival in Liberia.

The reproduction number for Ebola after initiation of intervention declined significantly, from an average of 1.7 to 0.1 secondary cases infected. The reproduction number measured before the start of interventions was similar to that reported for Liberia during the early phase of the epidemic before most interventions, including isolation facilities at ETUs and safe burials, were widely available (*15*). With an average of only 0.1 secondary infections per case after the public health responses began, the outbreaks terminated rapidly. A study in Conakry, Guinea, that linked 152 Ebola cases in transmission chains found a significant decline in the reproduction number from 2.3 in March before the start of interventions to 0.7 for case-patients admitted to ETUs after interventions were implemented; the reproduction number for case-patients not admitted to ETUs did not decline significantly from the preintervention period, suggesting that ETUs were important in reducing transmission (*16*). We could not measure the impact of safe and hygienic burials on transmission, but increased admission of case-patients to ETUs clearly helped reduce Ebola transmission in the communities included in this report. However, this finding does not imply that we can attribute all transmission reduction in these outbreaks to outside intervention. Communities actively participated in the response and, in different times and places, took measures to protect themselves, including engaging in social distancing, washing hands, avoiding traditional burial practices, and sending patients for treatment outside the community. Although not measureable, community interventions most likely contributed to some of the decline in transmission reported during these outbreaks.

Infection of secondary cases was clustered among few persons; 6 source-patients, all of whom died in the community, accounted for more than half of the cases in this report. “Superspreader” events have been documented previously, although whether these case-patients had more contacts overall or more contacts during periods of higher viremia, such as during the terminal illness or after death (*1*,*16*), is not clear. Community deaths overall generated 93% of secondary infections in these outbreaks. In contrast to our results, attendance at funerals in urban Conakry accounted for only 6% of cases (*16*). Although Ebola transmission around death could be relatively more important in rural than in urban areas, the classification of funeral exposure used in the analysis by Faye et al. (*16*) is likely to have excluded many contacts around the terminal illness and preparation of the body for burial, which generally take place before the day of the funeral. Regardless, Ebola deaths in the community have the potential to cause substantial transmission, and illness and deaths associated with funeral attendance should be considered a critical trigger for investigations of possible Ebola transmission.

As in previous reports (*17*,*18*), the age distribution of case-patients in these outbreaks did not reflect the general population. In Liberia, children <15 years of age comprise 43% of the population (*19*) but accounted for only 21% of case-patients. This low percentage could be the result of underreporting of infections in children or variation in patterns by age of exposure, infection, and clinical manifestations. We believe that the intensive investigations of each outbreak in this report limited the likelihood that cases in children were missed, but we cannot exclude that possibility because children were more likely than adults to be buried secretly in Liberia (D. Allen, pers. comm.). During the Ebola outbreak in Kikwit, Zaire, in 1995, children were determined to be at lower risk for Ebola because they were less likely to be exposed to body fluids (*20*). This lower risk also might be the case in Liberia, where children do not typically provide care to sick family members or participate in traditional funeral rites (*21*).

Although children appeared to be at lower risk than adults for Ebola, we did not find their CFR to be lower. In Sierra Leone, Schieffelin et al. found a significantly higher CFR for hospitalized persons >45 years (94%) than for those <21 years of age (57%) (*18*). In Guinea, Sierra Leone, and Liberia, an analysis of all reported cases found the odds for death to be higher for persons >45 years of age than for those <45 years (odds ratio [OR] 2.5, 95% CI 1.8–3.5), but the odds of dying for persons <15 years and >15 years of age did not differ (OR 1.2, 95% CI 0.8–1.7) (*15*). Although we found the highest CFR for case-patients >60 years of age, this CFR did not differ significantly from that of any other age group. This finding might be a limitation of our sample size or might indicate that the higher CFR for persons >45 years of age from other studies resulted from ascertainment bias arising from inclusion of only hospitalized case-patients or those reported to the surveillance system, whereas ascertainment of cases in this report was community-based.

Our dataset is subject to some important limitations. The primary objectives of the teams responding to the outbreaks were to facilitate patient care and interrupt Ebola transmission. The teams constructed transmission chains during outbreak responses primarily to identify any previously unrecognized case-patients or contacts who might continue to transmit Ebola in the community or spread it to other areas of the country; use of these data for epidemiologic analyses was a secondary priority. As a result, some data are missing, particularly critical dates, and there is most likely some inaccuracy in the data collected from proxies when the case-patient had died or left the area. These limitations are inherent to all datasets from this and similar epidemics when urgent response is the primary focus.

The data we present provide strong evidence that when capacity for isolation and treatment of Ebola is sufficient, rapid response strategies in remote areas that engage communities to promptly isolate and remove case-patients for care have the dual benefit of contributing to interruption of transmission and improving survival rates through treatment at ETUs. Provided basic interventions are implemented and communities are accepting, outbreaks of Ebola in rural areas can be controlled rapidly.

Technical Appendix Epidemiologic curves of Ebola cases by outbreak and location, incubation periods for Ebola cases, distribution of case-patients by time to different events or outcomes, distribution of 92 case-patients by length of stay in an Ebola treatment unit, and plot of the Schoenfeld residuals for Ebola treatment unit admission by time, Liberia, August–December 2014.
