# Supplementary material for: Decreased Ebola Transmission after Rapid Response to Outbreaks in Remote Areas, Liberia, 2014
Source: Emerg Infect Dis. 2015 Oct;21(10):1800–7. doi: 10.3201/eid2110.150912 (PMC4593457; doi:10.3201/eid2110.150912)
Supplement: Technical Appendix — Epidemiologic curves of Ebola cases by outbreak and location, incubation periods for Ebola cases, distribution of case-patients by time to different events or outcomes, distribution of 92 case-patients by length of stay in an Ebola treatment unit, and plot of the Schoenfeld residuals for Ebola treatment unit admission by time, Liberia, August–December 2014. [file 15-0912-Techapp-s1.pdf]

Article DOI: http://dx.doi.org/10.3201/eid2110.150912

Decreased Ebola Transmission after Rapid Response to Outbreaks in Remote Areas, Liberia, 2014

Technical Appendix

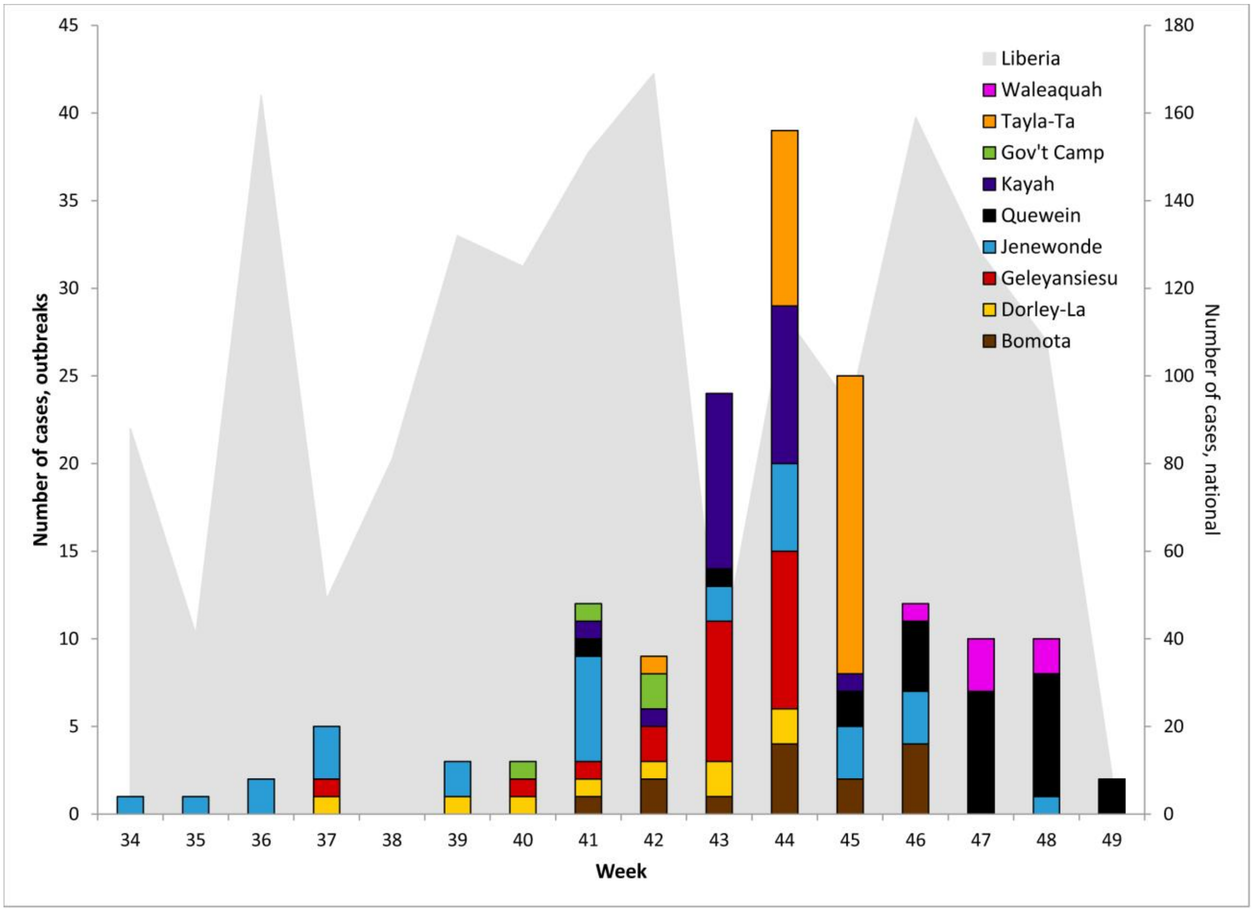

Technical Appendix Figure 1. Epidemiologic curve of Ebola virus disease cases, by outbreak, remote rural areas, Liberia, August–December 2014.

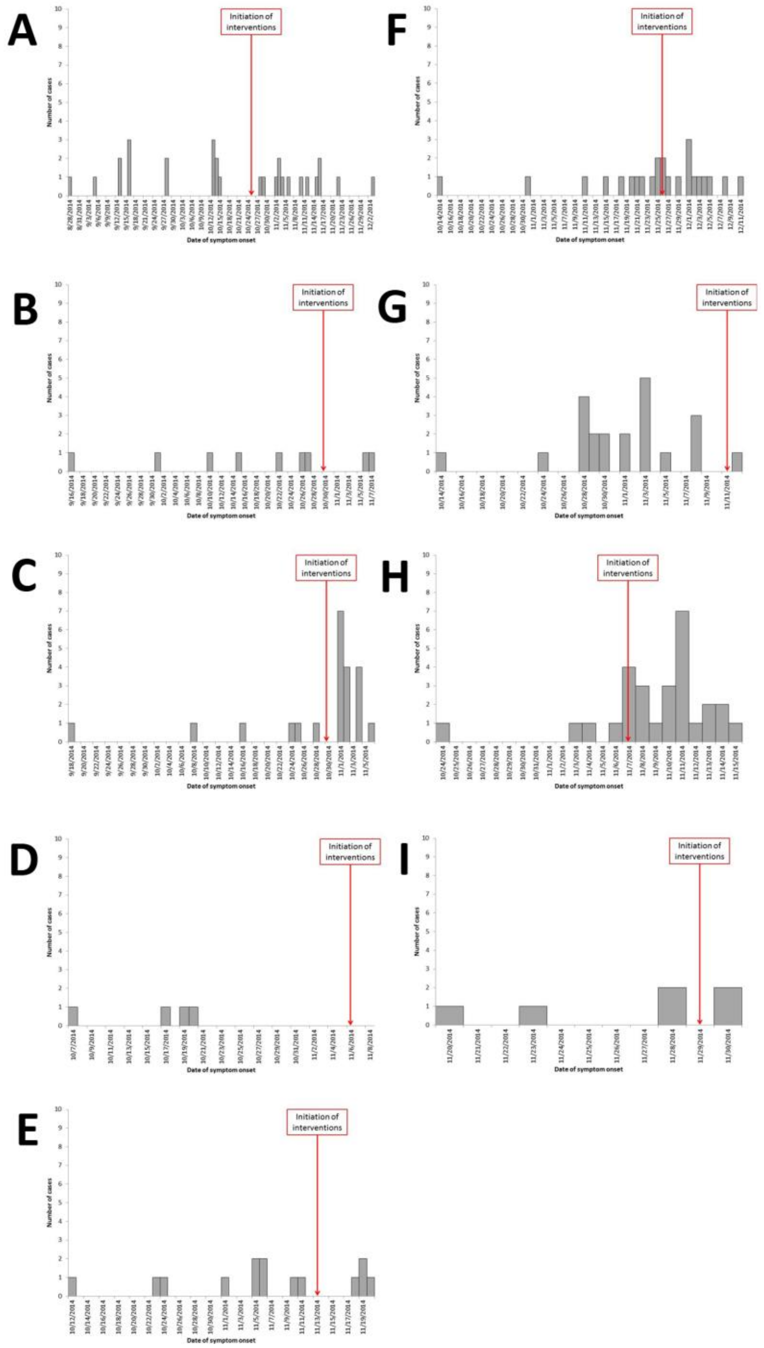

Technical Appendix Figure 2. Epidemiologic curves of Ebola virus disease cases, by location, remote rural areas, Liberia, August–December 2014. A) Jenewonde, Grand Cape Mount County. B) Dorley-La, Bomi County. C) Geleyansiesu, Gbarpolu County. D) Government Camp, Sinoe County. E) Bomota, Bong County. F) Quewein, Grand Bassa County. G) Kayah, Rivercess County. H) Tayla-Ta, Bong County. I) Waleaquah, Grand Cape Mount.

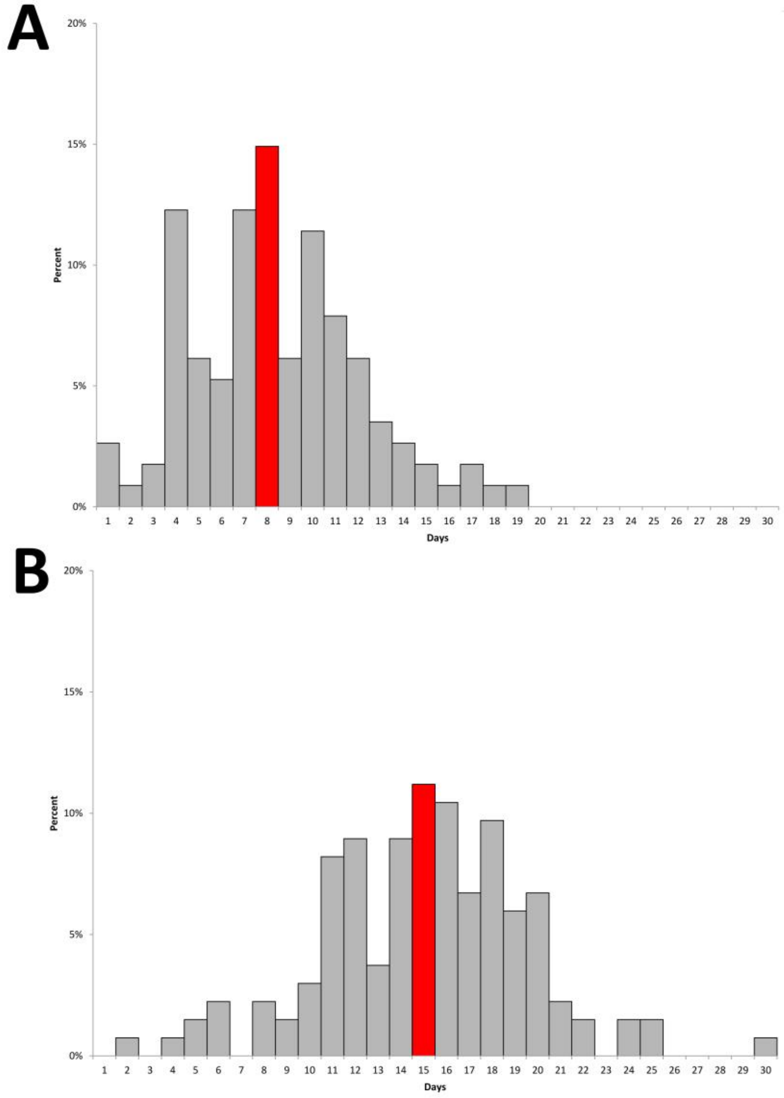

Technical Appendix Figure 3. Incubation periods for Ebola virus disease cases, remote rural areas, Liberia, August–December 2014. A) Minimum incubation period (n = 114). B) Clinical serial interval (n = 134). Red bars indicate the median value.

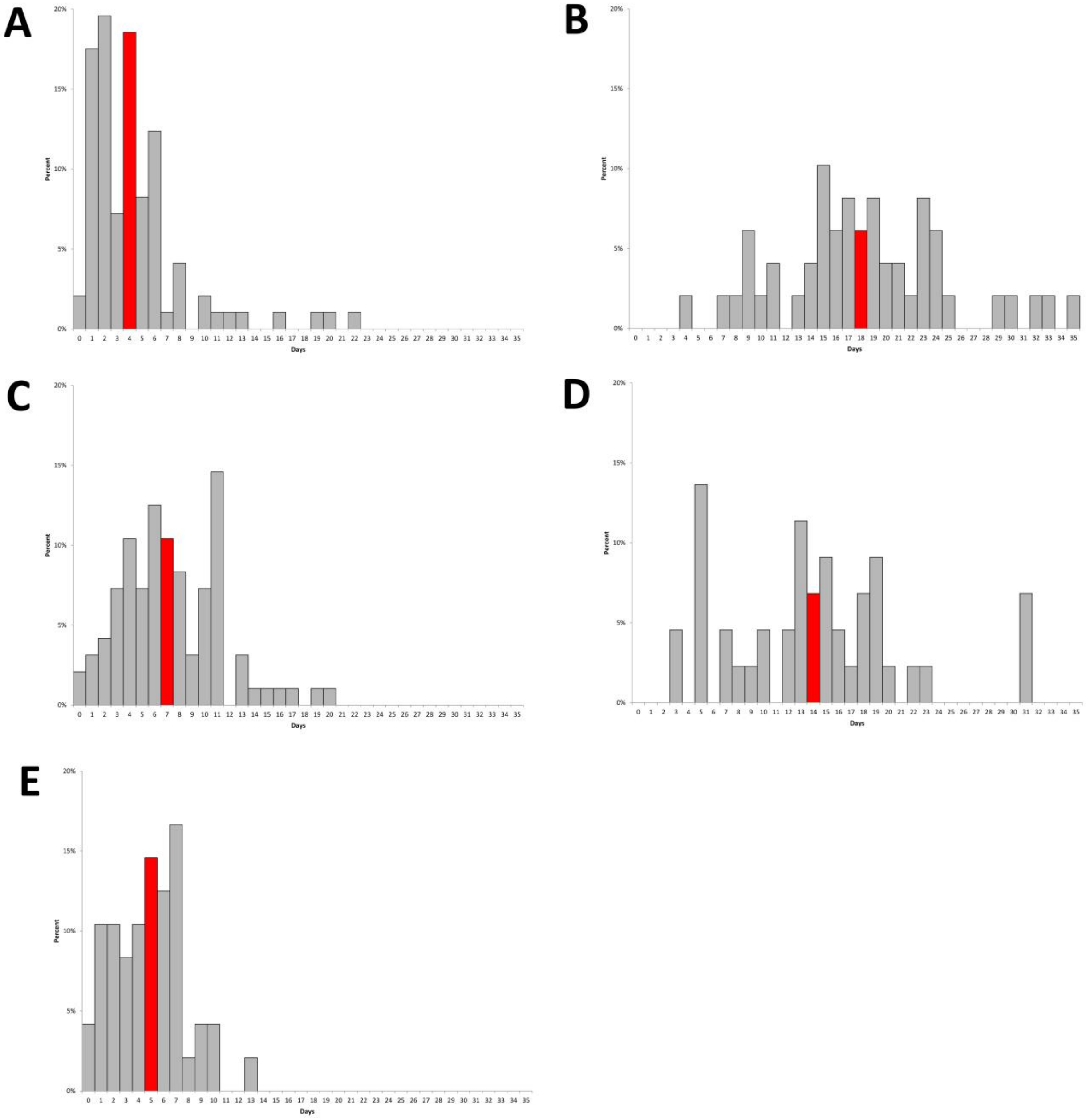

Technical Appendix Figure 4. Distribution of Ebola virus disease case-patients by time to different events or outcomes during outbreaks in remote rural areas, Liberia, August–December 2014. A) Number of days between symptom onset and admission (n = 97). B) Number of days from symptom onset to recovery (n = 49). C) Number of days from symptom onset to death (n = 97). D) Number of days from admission to recovery (n = 44). E) Number of days from admission to death (n = 48). Red bars indicate the median value.

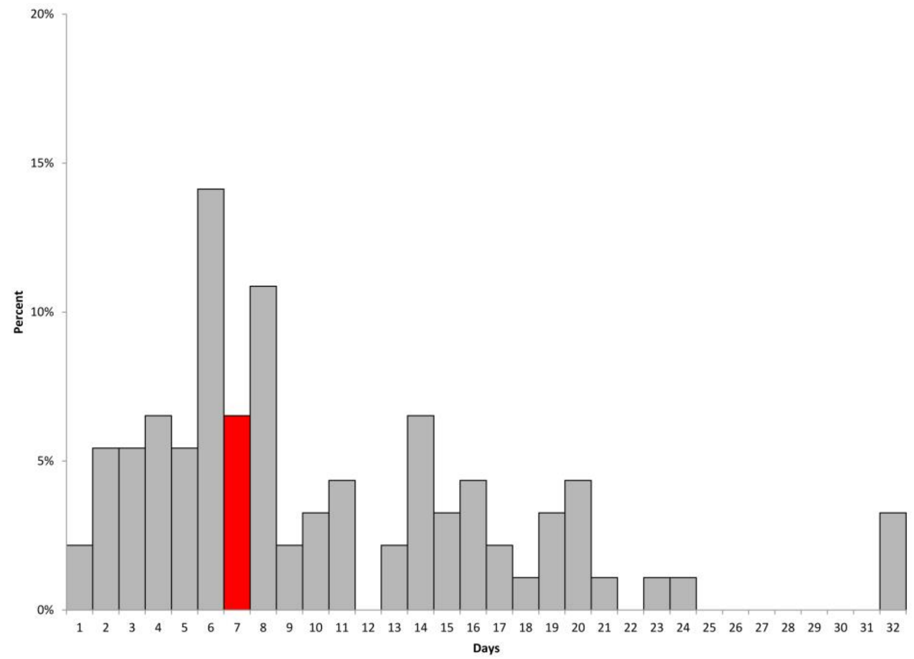

Technical Appendix Figure 5. Distribution of 92 Ebola virus disease case-patients, by length of stay in an Ebola treatment unit during outbreaks in remote rural areas, Liberia, August–December 2014.

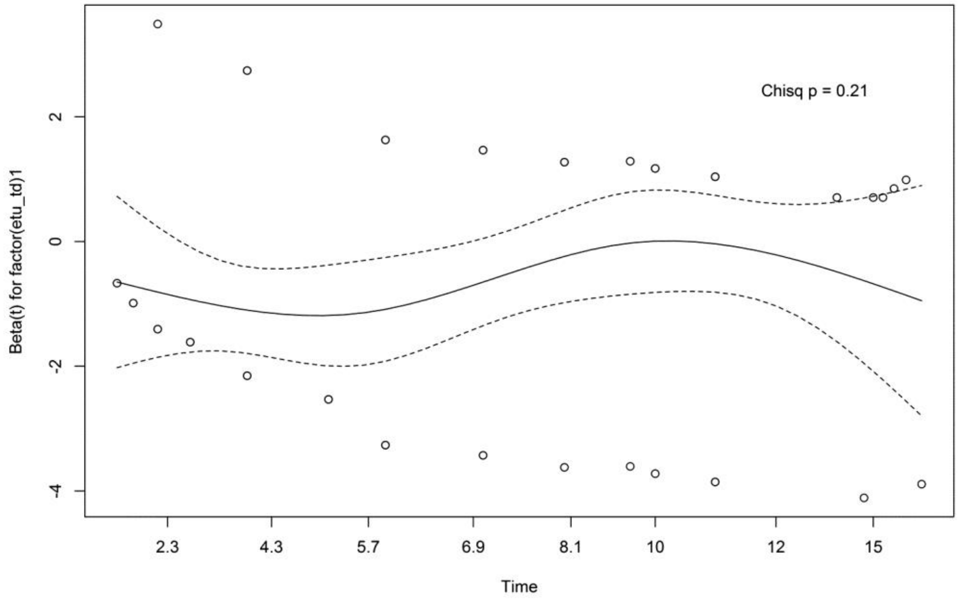

Technical Appendix Figure 6**.** Plot of the Schoenfeld residuals for Ebola treatment unit (ETU) admission by time, remote rural areas, Liberia, August–December 2014. The counting process method of accounting for time-dependent covariates is described in “Using time dependent covariates and time dependent coefficients in the Cox model” by Therneau and Crowson (2014 Jan 22, obtained from

http://cran.r-project.org/web/packages/survival/vignettes/timedep.pdf on 2014 Dec 23). Specifically, we used the [start,stop] form of data. A person admitted to the ETU would have 2 records, e.g., the following person had onset date of November 11, was admitted on November 15, and was discharged alive on November 28.

ID	etu	Time1	Time2	Event
29	0	0	3	0
29	1	3	17	0

Two persons were admitted on the date of symptom onset. They had a single record indicating etu = 1 and time1 = 0. Sixteen persons were admitted 1 day after symptom onset. This were given 2 records, the first having time1 = 0 and time2 = 0.5.
